# The association between vitamin D status and inflammatory bowel disease among children and adolescents: A systematic review and meta-analysis

**DOI:** 10.3389/fnut.2022.1007725

**Published:** 2023-01-09

**Authors:** Somaye Fatahi, Naseem Alyahyawi, Naryman Albadawi, Farzaneh Mardali, Naghi Dara, Mohammad Hassan Sohouli, Kousalya Prabahar, Pejman Rohani, Nazanin Koushki, Aliakbar Sayyari, Amir Hossein Hosseini, Ahmed Abu-Zaid

**Affiliations:** ^1^Pediatric Gastroenterology, Hepatology, and Nutrition Research Center, Research Institute for Children's Health, Shahid Beheshti University of Medical Sciences, Tehran, Iran; ^2^Department of Nutrition, School of Public Health, Iran University of Medical Sciences, Tehran, Iran; ^3^Department of Pediatrics, King Abdulaziz University Hospital, Jeddah, Saudi Arabia; ^4^Department of Medicine, King Faisal Specialist Hospital and Research Center, Riyadh, Saudi Arabia; ^5^Student Research Committee, Department of Clinical Nutrition and Dietetics, Faculty of Nutrition and Food Technology, Shahid Beheshti University of Medical Sciences, Tehran, Iran; ^6^Department of Pharmacy Practice, Faculty of Pharmacy, University of Tabuk, Tabuk, Saudi Arabia; ^7^Pediatric Gastroenterology and Hepatology Research Center, Pediatrics Centre of Excellence, Children's Medical Center, Tehran University of Medical Sciences, Tehran, Iran; ^8^College of Medicine, Alfaisal University, Riyadh, Saudi Arabia

**Keywords:** vitamin D, inflammatory bowel disease (IBD), children, systematic review, supplement

## Abstract

**Aim:**

Vitamin D deficiency is very common among children with IBD. Since there are conflicting results regarding the association of vitamin D with IBD, we conducted this systematic review to confirm the association of vitamin D with IBD.

**Methods:**

We conducted a systematic search in Scopus, Cochrane Library, Web of Science, PubMed, and Google Scholar to find relevant studies. Articles with cross-sectional and case-control designs that reported the association between vitamin D and IBD among children were included.

**Results:**

Eventually, 9 studies (with 16 effect sizes) reported the mean and SD or the median and the interquartile range of serum vitamin D levels in both subjects with IBD and control subjects. The random effects meta-analysis revealed that subjects with IBD had −1.159 ng/ml (95% CI: −2.783, 0.464) lower serum vitamin D concentrations compared with their healthy counterparts, but this difference was not significant. A total of 14 studies (with 18 effect sizes) with 2,602 participants provided information for the prevalence of vitamin D deficiency or insufficiency in patients with IBD as 44% (95% CI: 0.34–0.54) with significant heterogeneity noted among studies (*p* < 0.001; I^2^ = 97.31%).

**Conclusion:**

This systematic and meta-analysis study revealed that vitamin D deficiency was associated with IBD. Longitudinal studies should be conducted in the future to confirm our findings. Large randomized controlled trials assessing the doses of supplementation of vitamin D would provide a better understanding of the association between vitamin D and IBD.

## Introduction

Inflammatory bowel disease (IBD) includes both ulcerative colitis (UC) and Crohn's disease (CD). IBD is a systemic, chronic disease. UC is confined to the rectum and/or the colon, whereas CD involves the entire gastrointestinal tract, with the most common occurrence in the ileum and the colon (1). Overall, the pediatric prevalence of IBD increased by 133% from 2007 to 2016 in the United States, and the subgroup of children aged 10–17 years was the major contributor to the rising pediatric IBD prevalence (2). IBD presents differently in adults and children. For example, CD is more prevalent in pediatrics when compared to UC. This is completely different from that of adult IBD (3). Even among the two age groups, the disease characteristics differ. The complications are more in pediatric IBD compared to adults (4).

The exact etiology of IBD may be attributed to changes in the intestinal flora, residing in urban areas, and diets having high amounts of fats and carbohydrates (5). Various studies reported the role of vitamin D in IBD (6, 7). Vitamin D deficiency may lead to a reduction in bacterial clearance in the colon (8). This vitamin changes the immune responses by influencing macrophages and T lymphocytes, hence avoiding excessive immune responses, and also repairs the intestinal mucosal barrier (9, 10).

Low vitamin D levels are more common in patients with IBD (11). However, it is not specific whether low vitamin D levels are related to malabsorption due to damage in the intestinal mucosa (12). Some observational studies reported low vitamin levels in patients with IBD (13, 14), and some studies reported a lack of decrease in vitamin D levels (15, 16). Vitamin D deficiency is very common among children with CD. Even osteoporosis and growth retardation are commonly noticed in CD when compared to UC (17). Similar to our study, a study was conducted previously by Del Pinto et al. (18) in 2015, considering all age groups. However, focusing on the age group of children and adolescents is necessary due to their critical age for growth and development. In addition, no significant results were obtained in that subgroup analysis due to the limitations of studies related to children and adolescents. Since there is no clear evidence of a direct association between vitamin D and IBD among children and adolescents, we conducted this systematic review to confirm the association of vitamin D with IBD.

## Materials and methods

In this systematic review and meta-analysis, we investigated the data extracted based on components of the PRISMA (Preferred Reporting Items for Systematic Reviews and Meta-Analyses) guidelines (19) and MOOSE (Meta-Analyses of Observational Studies in Epidemiology) checklist (20) on the relationship between serum vitamin D and inflammatory bowel disease in pediatric patients.

### Search strategy and selection process

A structural and comprehensive literature was accomplished based on articles published up to 20 February (year) in the following electronic databases: Scopus, Cochrane Library, Web of Science, PubMed, and Google Scholar. The search used the terms “vitamin D,” “ergocalciferol,” “inflammatory bowel disease,” “Crohn's disease,” and “ulcerative colitis” in several combinations. The full electronic search strategy is reported in Supplementary Table 1. There were no date or language restrictions on imported articles. Moreover, all clinical trial and review article references were checked to find any relevant studies. After deleting duplicate articles, the title and abstract screening processes were performed. Subsequently, two researchers studied the full-text papers independently to find all the obligatory data for meta-analysis. The third researcher was called to resolve any discrepancies. Only articles that met the following criteria were included in this meta-analysis:

a) Observational studies—either case-control, cross-sectional, or cohort designs,
b) Studies with adolescents or children (age < 18 years),
c) Articles that showed mean and standard deviation (SD) or prevalence of insufficiency or deficiency of serum vitamin D levels in patients with IBD (Crohn's disease or ulcerative colitis).

Papers with (1) randomized clinical trial designs, (2) animal-based studies, (3) gray literature (chapters of books, abstracts in conferences, or review articles), (4) adult patients, (5) athletics, and (6) assessment of IBD in the acute phase were excluded from the analysis.

### Data extraction and syntheses

Required information of the eligible papers was extracted by two observers, such as the author's last name, publication date, the study location, study design, mean age of cases and controls, the number of cases and controls, type of study population (UC or CD), mean and SD of vitamin D concentration in patients and healthy participants or prevalence of vitamin D deficiency or insufficiency status, seasonally matching or adjusting, and method of vitamin D assessment.

### Quality assessments

The quality of the chosen papers in this meta-analysis was assessed using the Newcastle-Ottawa scale (21), which included 9 questions in three main sections such as selection of participants (0–4 points), comparability between groups (0–3 points), and outcome assessment (0–3 points). The quality evaluation process was reviewed by two investigators, and any differences in scoring were resolved by consensus.

### Statistical analyses

All data analyses were performed using the Stata 13 software (Stata Corp., College Station, Texas, USA). Data on vitamin D levels in patients with IBD in case-control studies were reported as weighted mean differences and SD or prevalence of deficiency or insufficiency in cross-sectional articles. The studies were conducted in different populations and countries, so the random-effects analysis was used to control variation. To determine the heterogeneity, the I-square (I^2^) index was assessed by random-effects analysis, and high heterogeneity was described if I^2^ was more than 50%. A sensitivity analysis was conducted to find out which study had the highest proportion in the pool effect size. Afterward, Egger's regression model was used to examine the publication bias in the funnel plots. Due to the high heterogeneity between records, we decided to carry out the subgroup analysis to distinguish the possible reasons for high heterogeneity and the effect of variant agents on the relationship between serum levels of vitamin D and IBD. These subgroups were based on assessing vitamin D methods such as HPLC (high-performance liquid chromatography), RIA (radioimmunoassay), and chemiluminescence-based competitive protein-binding assay and evaluating matching the season to vitamin D concentration (yes, matched; NR, not reported).

### Literature review

**Figure 1:**
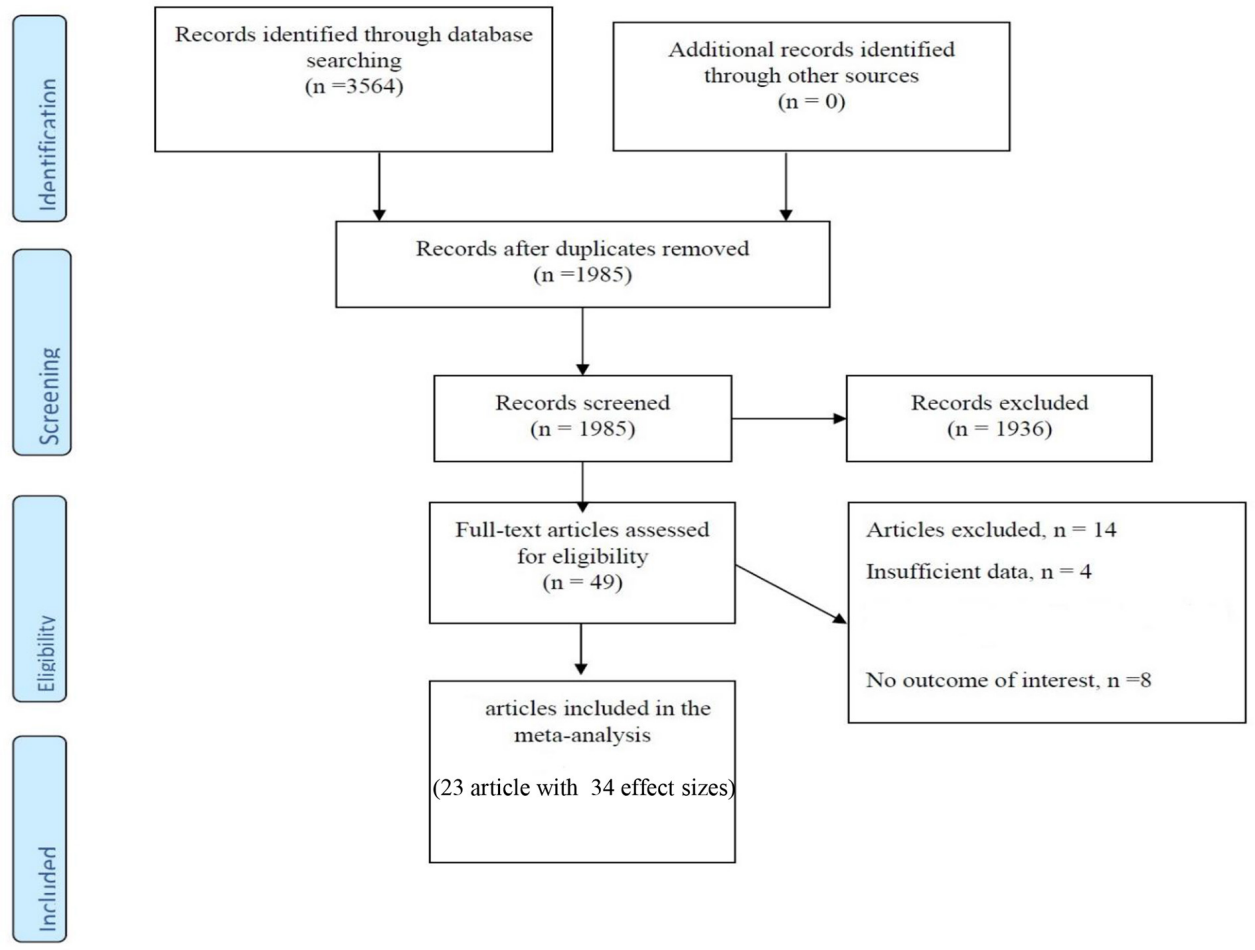
Flow chart of the included studies, including identification, screening, eligibility and the final sample included.

The main characteristics of case-control and cross-sectional studies are presented in Table 1 and Figure 1. Approximately 3,564 articles were searched electronically; 1,579 duplicate items were removed in the screening process, and 1,936 articles were eliminated as they had no relation to the purpose of the study. In addition, 14 articles did not meet the qualitative and quantitative criteria given in this study. Notably, a related cohort study was identified, which was omitted due to the format of the analysis, so we described it in the discussion part. Eventually, 9 articles (16 effect sizes) with a case-control design declared the concentration of vitamin D in children with a mean (SD), and 14 studies (with 18 effect sizes) declared the prevalence of vitamin D deficiency or insufficiency in pediatrics. These studies were published between 2003 and 2019, and most of the records (*n* = 11) were conducted in the United States (22, 25, 29–31, 33–36, 38, 39, 42, 43) and the rest in Canada (23, 40), Australia (32), Finland (24), Denmark (26), Italy (28), South Korea (27, 41), and Israel (37). In general, we entered data of 4,803 children participants with an age range of 2–18 years. Based on the methodological assay, the quality score was at least 6 points for the articles, and the majority gained high-quality points. Notably, the vitamin D measurement seasons were different in the studies, and some did not report any data, and some case-control studies presented season matching or adjustment. In addition, various measurement methods have been applied; some studies used HPLC (high-performance liquid chromatography) (24, 43) or RIA (radioimmunoassay) (29) and others applied chemiluminescence-based competitive protein-binding assays (23, 28, 30, 32, 35, 36, 39, 44) to measure the concentration of vitamin D.

**Table 1:** Characteristics of studies.

**No**	**References**	**Country**	**Study Design**	**Population of study**	**Mean age, y**	**Total N**	**N case**	**Mean/ sdcase (ng/ml)**	**N control**	**Mean/sd control (ng/ml)**	**Deficiency or insufficiency status**	**Season adjust/ matching**	**Season of measurement**	**Q**	**Method of vitamin D assessing**
1	Nwosu (22)	USA	Case-control	CD	8.5		25	28.24+10.28	49	26.16 + 10.44		NR/yes	Winter + spring,14/25 (56)		DiaSorin Liaison
2	El-Matary et al., (23)	Canada	Case-control	CD	12.2		39	26.68 + 10.2	56	32.68 + 6.16		NR/yes	NR		Protein binding assay after column chromatography (Esoterix
3	El-Matary et al. (23)	Canada	Case-control	UC	12.2		21	22.76 + 8.8	56	32.68 + 6.16		NR/yes	NR		
4	Laakso et al. (24)	Finland	Case-control	IBD	14.9		80	21.6 + 25.48	80	18.5 + 19.25		NR/ NR	NR		(HPLC)
5	Alkhouri et al. (15)	USA	Case-control	CD	12.3		46	29.9 + 12.7	61	26.7 + 9.4		NR/ NR	Is less vitamin D deficiency in the summer months as compared with		(HPLC)
6	Alkhouri et al. (15)	USA	Case-control	UC	12.3		12	32 + 25.8	61	26.7 + 9.4		NR/ NR	Spring (P$\frac{1}{4}$0.08), autumn (P$\frac{1}{4}$0.15), and winter (P$\frac{1}{4}$0.08).		
7	Alkhouri et al. (15)	USA	Case-control	ID	12.3		3	21.7 + 7.5	61	26.7 + 9.4		NR/ NR	Spring (P$\frac{1}{4}$0.08), autumn (P$\frac{1}{4}$0.15), and winter (P$\frac{1}{4}$0.08).		
8	Prosnitz (Black participants) (25 )	USA	Case-control	CD	13.5		8	10.5 + 4.6	62	15.8 + 7.9					Radioimmunoassay (RIA) with I125-labeled tracer
9	Prosnitz (Non-black participants) (25)	USA	Case-control	CD	13.5		70	23.5 + 0.2	159	25.3 + 8.7		Yes/NR	Winter		
10	Veit et al. (16)	USA	Case-control	IBD	16.4 Control (14.6)		58	24 + 10.24	116	24 + 10.88		Yes/yes	Summer–fall, %:57.6		DiaSorin Liaison
11	Thorsen et al. (26)	Denmark	Case-control	CD	14		155	11.2 + 6.54	384	10.68 + 6.9		NR/Yes	All		Liquid chromatography mass spectrometry (LC-MS)
12	Thorsen et al. (26)	Denmark	Case-control	UC	15		210	10.92 + 7.08	384	10.68 + 6.9		NR/Yes	All		
13	Thorsen et al. (26)	Denmark	Case-control	IC	11		19	12.72 + 8.68	384	10.68 + 6.9		NR/Yes	All		
14	Sohn et al. (27)	Korea	Case-control	CD	14.4		43	16.3 + 9.3	45	16.5 + 5.3		NR/NR	NR		Liquid chromatography mass spectrometry (LC-MS)
15	Sohn et al. (27)	Korea	Case-control	UC	14.4		17	19.9 + 7.2	45	16.5 + 5.3		NR/NR	NR		
16	Strisciuglio et al. (28)	Italy	Case-control	IBD	11 (Control: 9.4)		33	19.2 + 9.7	18	28.2 + 12.1		NR/Yes	Winter-spring		Enzyme-linked immunosorbent
17	Sentongo et al. (29)	Philadelphia	Cross-sectional	CD	15.76	113					16% < 20 ng/ml Hypovitaminosis < 38 nmol/L.				Radioimmunoassay with a radioiodinated tracer
18	Pappa et al. (30)	USA	Cross-sectional	IBD	15.76	130					34.6% < 15 ng/ml 10.8%: 25OHD ≤ 8				Nichols Advantage chemiluminescence-based competitive protein-binding assay (Nichols Institute Diagnostics,San Clemente, CA
19	Pappa et al. (30)	USA	Cross-sectional	CD	15.76	94					38.3% < 15 ng/ml 12.8%: 25OHD ≤ 8				Nichols Advantage chemiluminescence-based competitive protein-binding assay (Nichols Institute Diagnostics,San Clemente, CA
20	Pappa et al. (30)	USA	Cross-sectional	UC	15.76	36					25% < 15 ng/ml 5.6 %: 25OHD ≤ 8				Nichols Advantage chemiluminescence-based competitive protein-binding assay (Nichols Institute Diagnostics,San Clemente, CA
21	Salehi et al. (31)	USA	Cross-sectional	IBD	NR	110					63.6% < 20 ng/ml < 50 nmol/l				NR
22	Levin et al. (32)	Australia	Cross-sectional	IBD	12.6	263					19: 20–30 ng/ml 51–75 nmol/l				Antibody-based chemiluminescence assay
23	Levin et al. (32)	Australia	Cross-sectional	IBD	12.6	263					38 < 20 ng/ml Defficiency < 51 nmol/l				Antibody-based chemiluminescence assay
24	Goldberg et al. (33)	USA	Cross-sectional	UC	2-21	24					80 < 20 ng/ml Insufficiency 20–30 nmol/L				NR
25	Goldberg et al. (33)	USA	Cross-sectional	CD	2-21	52					60 < 20 ng/ml Insufficiency 20–30 nmol/L				NR
26	Schaefer et al. (34)	USA	Cross-sectional	IBD	NR	92					< 30 ng/mL < 30 ng/mL				NR
27	Syed et al. (35)	USA	Cross-sectional	IBD	5-18.9	69					77 20–30 ng/ml Insufficiency < 30 ng/mL				Automated chemiluminescent technique (Automated IDS-iSYS System, Immunodiagnostic Systems, Fountain Hills, AZ)
28	Syed et al. (36)	USA	Cross-sectional	IBD	5-18.9	69					38 < 20 ng/ml Deficiency < 20 ng/ml				Automated chemiluminescent technique (Automated IDS-iSYS System, Immunodiagnostic Systems, Fountain Hills, AZ)
29	Brandvayman et al. (37)	Israeil	Cross-sectional	IBD	14	623					21 < 20 ng/ml defficiency				NR
30	Winter et al. (38)	USA	Cross-sectional	IBD		203					31 < 20 ng/ml LOW < 9 ng/ml				NR
31	Sauer et al. (39)	USA	Cross-sectional	UC	12.7	388					57 20–30 ng/ml insufficient < 30 ng/Ml				IDS-iSYS 25-Hydroxyvitamin D automated chemiluminescense immunoassay (Gaithersburg, MD)
32	Mager et al. (40)	Canada	Cross-sectional	CD	NR	102					56.80% 20–30 ng/ml insufficiency < 50 nmol/l				Liquid chromatogrpahy-mass spectroscopy (LC-MS) technology
33	Mager et al. (40)	Canada	Cross-sectional	UC	NR	63					43.20% 20–30 ng/ml insufficiency < 50 nmol/l				Liquid chromatogrpahy-mass spectroscopy (LC-MS) technology
34	Kim et al. (41)	Korea	Cross-sectional	IBD	13.1	96					56.80% 20–30 ng/ml insufficiency < 50 nmol/l				Liquid chromatogrpahy-mass spectroscopy (LC-MS) technology

### Meta-analysis

**Figure 2:**
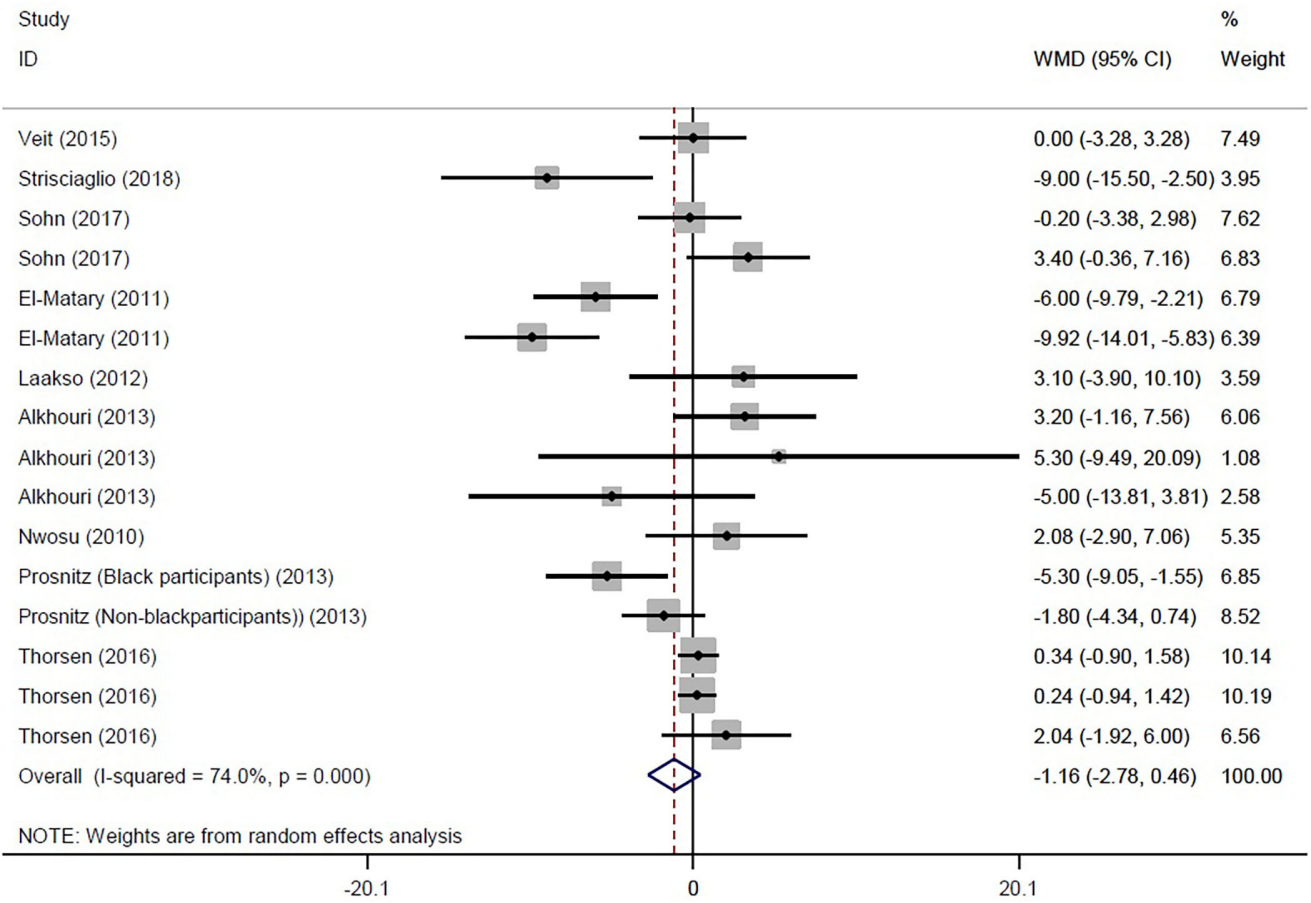
Forest plot show the WMD in serum vitamin D concentrations between participants with IBD and healthy control.

Eventually, 16 studies reported the mean and SD or median and interquartile range of serum vitamin D levels in both IBD and control subjects. The random effects meta-analysis revealed that subjects with IBD had −1.159 ng/ml (95% CI: −2.783, 0.464) lower serum vitamin D concentrations compared with their healthy counterparts, but this difference was not significant (Figure 2). However, the tests also showed that there was significant heterogeneity among the studies (*I*^2^ = 74.0%, *P* < 0.001). Heterogeneity in meta-analysis refers to the variation in study results between studies. To find out the source of heterogeneity, we conducted studies based on the following subgroups: type of patients (IBD, UC, CD, or IC), assessment method of 25OHD, and matching of the season for the control group. In the subgroup analysis, results remained non-significant, while the types of patients (IBD, UC, and CD) and assessment method of 25OHD were considered as possible sources of heterogeneity (Supplementary Figures 1–3).

**Figure 3:**
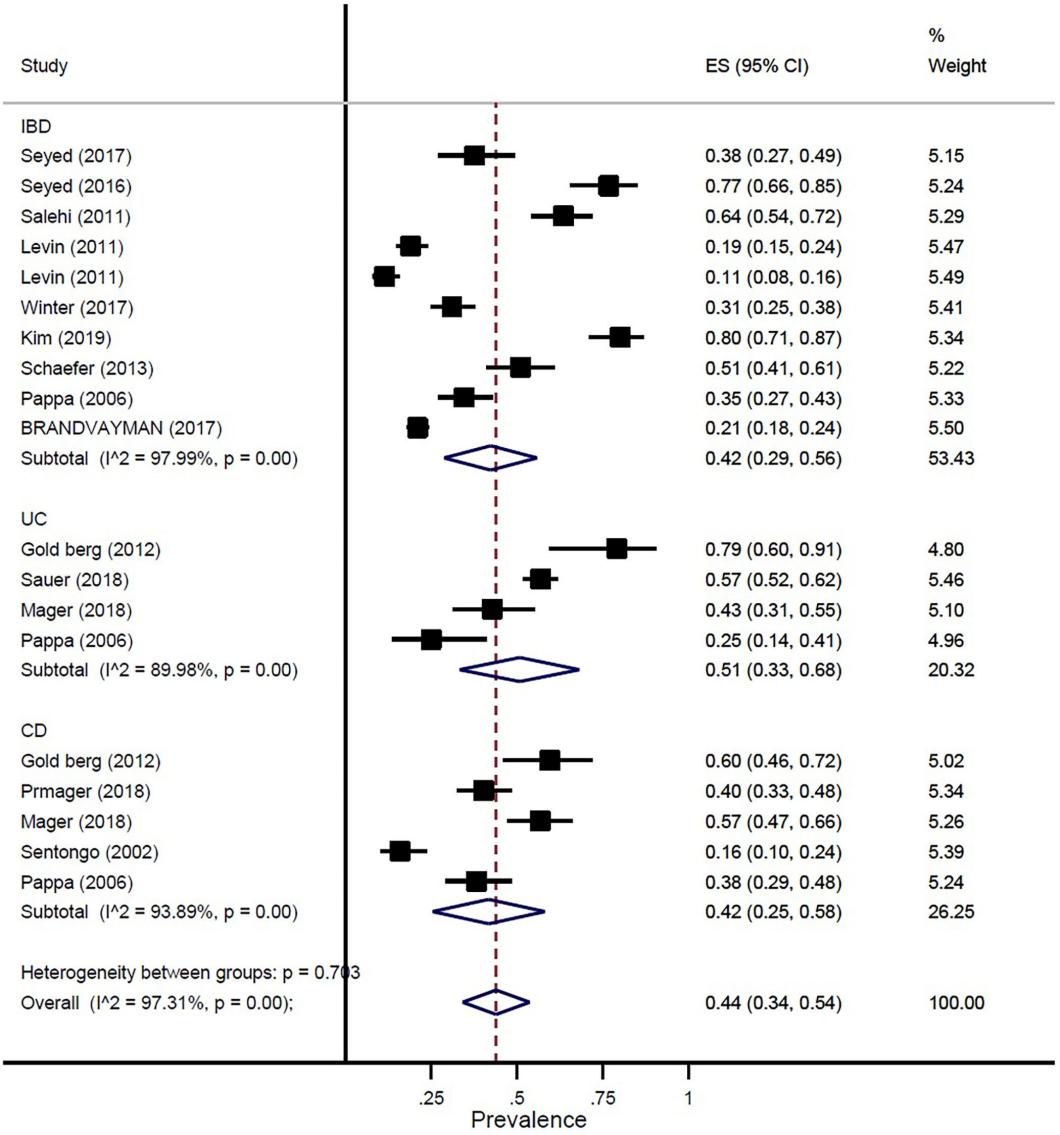
Pooled estimate of the prevalence of vitamin D deficiency or insufficiency in children and adolescence with IBD, UC, and CD.

**Figure 4:**
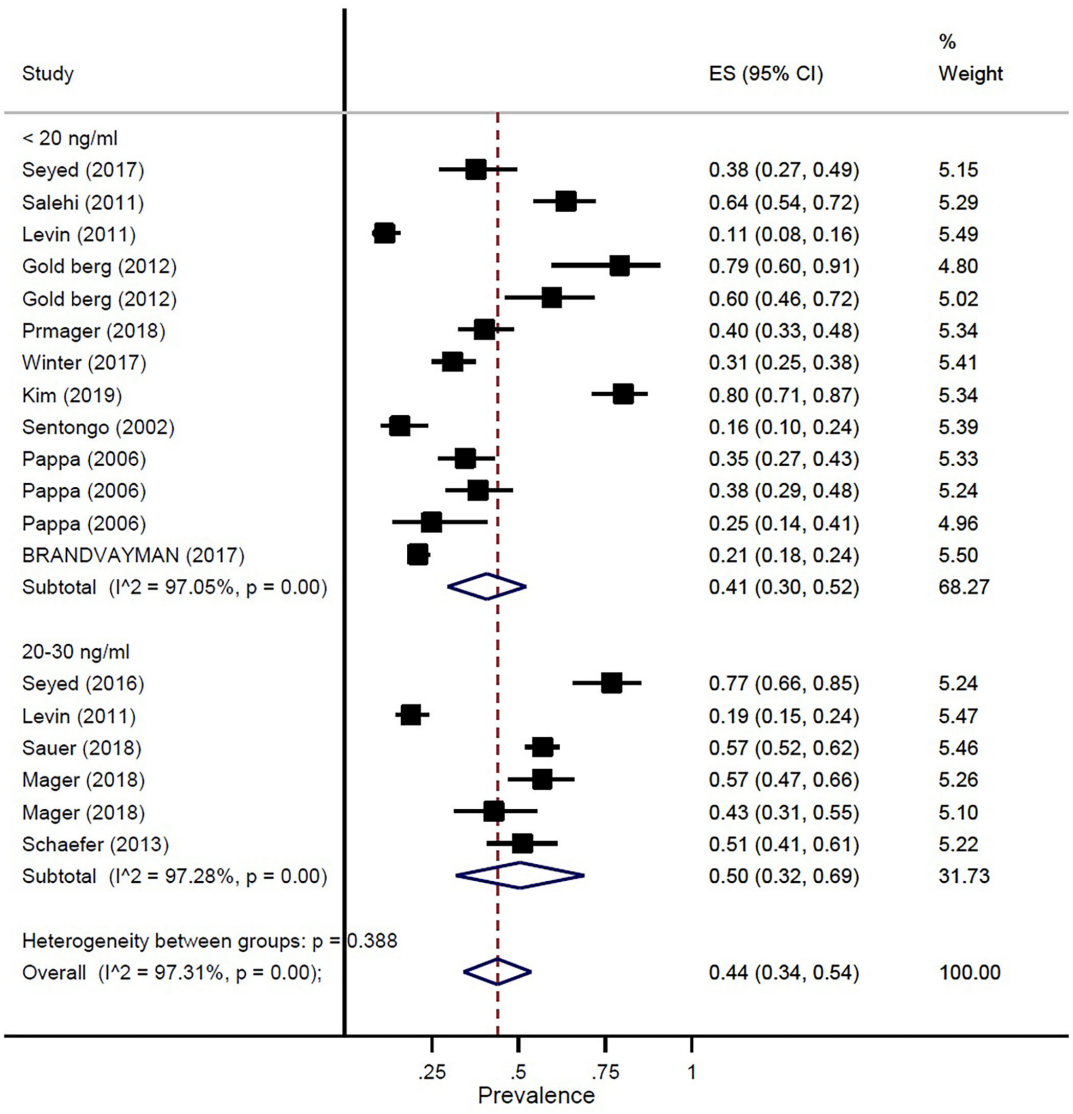
Pooled estimate of the prevalence of vitamin D status in children and adolescence with IBD.

The prevalence of vitamin D deficiency or insufficiency in patients with IBD was found as 44% (95% CI: 0.34–0.54) by eighteen studies with a total of 2,602 participants, with significant heterogeneity noted among studies (*p* < 0.001; I^2^ = 97.31%). The pooled prevalence of vitamin D deficiency or insufficiency in patients with IBD was 42% (95% CI: 0.29–0.56), UC was 51% (95% CI: 0.33–0.68), and CD was 42% (95% CI: 0.25–0.58) (Figure 3). In addition, the prevalence of participants with a level of vitamin D < 20 ng/ml was 41% (95% CI: 0.30–0.52) and the level of 20–30 ng/ml was 50% (95% CI: 0.32–0.69) (Figure 4).

### Publication bias and sensitivity analysis

Evaluation of publication bias by visual inspection of the funnel plot and Egger's test demonstrated the evidence for publication bias in the meta-analysis of the prevalence of vitamin D deficiency or insufficiency in patients with IBD (*p* = 0.05) (Supplementary Figure 4). However, the results of the meta-trim-and-fill analysis found no studies. Egger's linear regression test for case-control studies revealed no publication bias (*p* = 0.31) (Supplementary Figure 5). Sensitivity analysis revealed that removing some of the studies would not have a significant impact on the overall results (Supplementary Figures 6, 7).

## Discussion

This systematic review and meta-analysis were conducted using 35 articles. Our study results showed that vitamin D is deficient in children with IBD when compared to the healthy control group. But the results were not statistically significant. These results were similar to another study. A meta-analysis was conducted, including 14 studies of 1,891 patients, and reported that patients with IBD had more vitamin D deficiency when compared to controls, and the results were statistically significant (18). Vitamin D regulates cytokine secretion, which is involved in the inflammatory response in immune systems (45). Many studies that were conducted previously have reported that the levels of vitamin D are lower in children with IBD (23, 46). Even there are contradictory results regarding the association of vitamin D with IBD (15). Sufficient vitamin D has been associated with clinical benefits in children with IBD. There was a reduction in disease activity in children with sufficient vitamin D (41). Guzman-Prado et al. also showed that vitamin D supplementation in patients with IBD and vitamin D deficiency is effective at correcting vitamin D levels and is associated with improvements in clinical and biochemical disease activity scores (47).

The subgroup analysis of our study did not reveal a statistically significant deficiency in vitamin D concentrations. The same results were obtained in a study conducted by Kim (41). El-Matary et al. (23) reported that the levels of vitamin D were lower in children with UC, but it was not statistically significant. Another study reported that the levels of vitamin D were higher in children with CD, and it was statistically significant (48). Sensitivity analysis revealed that removing some of the studies would not have a significant impact on the overall results. The quality of all the studies was assessed using the Newcastle-Ottawa scale.

The scientific evidence on the role of vitamin D in children with IBD was reviewed by studies conducted previously (6, 49–51). Although, the effective vitamin D supplementation in children with IBD remains controversial. Our meta-analysis evaluating vitamin D status in 2602 participants, a large cohort population, would provide consistent and useful results.

A low level of vitamin D is clinically significant. Both UC and CD are related to environmental factors (52). Hypovitaminosis D in IBD is related to malabsorption, which may be due to inflammation in the bowel or surgical resection (12); less exposure to sunlight (50); and higher intake of vitamin D by the inflammatory cells (53).

In our study, the prevalence of vitamin D deficiency or insufficiency in patients with IBD was 44%. The pooled prevalence of vitamin D deficiency or insufficiency in patients with IBD was 42%, UC was 51%, and CD was 42%. In addition, the prevalence of participants with a level of vitamin D of < 20 ng/ml was 41% and the level of vitamin D of 20–30 ng/ml was 50%. Our study results confirmed prior study findings that reported a high prevalence of vitamin D deficiency in IBD (13, 29).

There are a few major strengths of the present systematic review and meta-analysis. First, a large number of studies were included, which provided a large sample size. In addition, we successfully pooled the results of subgroup analyses, sensitivity analyses, and publication bias. Similarly, there are some limitations in our study that should be considered. First, there existed a substantial degree of heterogeneity in the designs of studies, with a wide range of methods of vitamin D assessment. Nonetheless, we performed subgroup analysis to find possible sources of heterogeneity. Another important limitation of this study is that we could not identify the relationship between the season of vitamin D level measurement and IBD because many studies did not report this variable well.

## Conclusion

This systematic review and meta-analysis study showed that patients with IBD were associated with vitamin D deficiency. Longitudinal studies should be conducted in the future to confirm our findings. Large randomized controlled trials assessing the doses of vitamin D supplementation would provide a better understanding of the association between vitamin D and IBD.

## Data availability statement

The original contributions presented in the study are included in the article/Supplementary material, further inquiries can be directed to the corresponding authors.

## Author contributions

SF, NAly, NAlb, FM, and MS contributed to the conception, design, and statistical analysis. SF, KP, NAly, NAlb, FM, PR, NK, AH, and AS contributed to data collection and the manuscript draft. AS, AH, and AA-Z supervised the study. All authors contributed the manuscript draft, critical revision, and approved the final version of the manuscript.
