# Supplementary material for: The association between vitamin D status and inflammatory bowel disease among children and adolescents: A systematic review and meta-analysis
Source: Front Nutr. 2023 Jan 9;9:1007725. doi: 10.3389/fnut.2022.1007725 (PMC9868587; doi:10.3389/fnut.2022.1007725)
Supplement: Supplementary file 1 [file Table_1.docx]

| Supplementary table 1. Search Strategies  PubMed |
| --- |
| ("vitamin d"[MeSH Terms] OR "vitamin d"[All Fields] OR "ergocalciferols"[MeSH Terms] OR "ergocalciferols"[All Fields]) OR ("25-hydroxyvitamin D"[Supplementary Concept] OR "25- hydroxyvitamin D"[All Fields] OR "25 hydroxyvitamin d"[All Fields] OR "calcifediol"[MeSH Terms] OR "calcifediol"[All Fields]) OR (25[All Fields] AND ("hydroxide ion"[Supplementary Concept] OR "hydroxide ion"[All Fields] OR "oh"[All Fields]) AND ("vitamins"[Pharmacological Action] OR "vitamins"[MeSH Terms] OR "vitamins"[All Fields] OR "vitamin"[All Fields]))) AND (("colitis, ulcerative"[MeSH Terms] OR ("colitis"[All Fields] AND "ulcerative"[All Fields]) OR "ulcerative colitis"[All Fields] OR ("ulcerative"[All Fields] AND "colitis"[All Fields])) OR ("crohn disease"[MeSH Terms] OR ("crohn"[All Fields] AND "disease"[All Fields]) OR "crohn disease"[All Fields] OR ("crohn's"[All Fields] AND "disease"[All Fields]) OR "crohn's disease"[All Fields]) OR ("inflammatory bowel diseases"[MeSH Terms] OR ("inflammatory"[All Fields] AND "bowel"[All Fields] AND "diseases"[All Fields]) OR "inflammatory bowel diseases"[All Fields] OR ("inflammatory"[All Fields] AND "bowel"[All Fields] AND "disease"[All Fields]) OR "inflammatory bowel disease"[All Fields])) AND "Child OR "Adolescent OR "Pediatrics"[Mesh] OR Pediatric* OR youth* OR teen*. |
