# Supplementary material for: The association between vitamin D status and inflammatory bowel disease among children and adolescents: A systematic review and meta-analysis
Source: Front Nutr. 2023 Jan 9;9:1007725. doi: 10.3389/fnut.2022.1007725 (PMC9868587; doi:10.3389/fnut.2022.1007725)
Supplement: Supplementary file 2 [file Data_Sheet_1.DOCX]

**Supplementary Figure1**. Forest plot show the WMD in serum vitamin D concentrations between participants with IBD (UC,CD and IC) and healthy control.

**Supplementary Figure2**. Forest plot show the WMD in serum vitamin D concentrations between participants with IBD and healthy control by assessment method of 25OHD.

**Supplementary Figure3**. Forest plot show the WMD in serum vitamin D concentrations between participants with IBD and healthy control by matching of season for controls.

|  |  |
| --- | --- |
| **Supplementary figures 4.** Funnel plot of pooled analysis for prevalence of vitamin D deficiency or insufficiency in IBD patients | **Supplementary figures 5.** Funnel plot of the weighted mean difference of vitamin D level in IBD patients. |

|  |  |
| --- | --- |
| **Supplementary figure 6, 7.** Sensitivity analysis observed no significant effect of any individual study on the combine effect sizes. | |
